# The importance of dysphagia screening and nutritional assessment in hospitalized patients

**DOI:** 10.1590/S1679-45082018AO4189

**Published:** 2018-05-25

**Authors:** Patrícia Amaro Andrade, Carolina Araújo dos Santos, Heloísa Helena Firmino, Carla de Oliveira Barbosa Rosa

**Affiliations:** 1 Universidade Federal de Viçosa, Viçosa, MG, Brazil; 2 Hospital São Sebastião, Viçosa, MG, Brazil

**Keywords:** Deglutition disorders, Nutrition assessment, Malnutrition, Hospitals, Transtornos de deglutição, Avaliação nutricional, Desnutrição, Hospitais

## Abstract

**Objective:**

To determine frequency of dysphagia risk and associated factors in hospitalized patients as well as to evaluate nutritional status by using different methods and correlate the status with scores of the Eating Assessment Tool (EAT-10).

**Methods:**

This was a cross-sectional study including 909 inpatients of a philanthropic hospital. For the diagnosis of dysphagia we used an adapted and validated Brazilian version of the Eating Assessment Tool (EAT-10). The nutritional status was evaluated through the subjective global assessment, and anthropometric measurements included weight, calf and arm circumference, and knee height. The Mann-Whitney test, associations using the Pearson’s χ^2^ and Spearman’s correlation were used to verify differences between the groups.

**Results:**

The prevalence of dysphagia risk was 10.5%, and aging was the associated factor with this condition. Patients at risk presented lower values of arm and calf circumference, variables that correlated inversely with the Eating Assessment Tool (EAT-10) score. Malnutrition was observed in 13.2% of patients based on the subjective global assessment and in 15.2% based on the Body Mass Index.

**Conclusion:**

Screening for dysphagia and malnutrition should be introduced in hospitals routine to avoid or minimize damages caused by dysphagia or malnutrition, especially among older people.

## RESUMO

**Objetivo:**

Verificar a prevalência do risco de disfagia e seus fatores associados em pacientes hospitalizados, bem como avaliar o estado nutricional por diferentes métodos e correlacioná-los à pontuação do *Eating Assessment Tool* (EAT-10).

**Métodos:**

Estudo de delineamento transversal, realizado com 909 pacientes internados em um hospital filantrópico. Para o rastreamento de disfagia, foi aplicado o *Eating Assessment Tool* (EAT-10) em sua versão adaptada para o Brasil. O diagnóstico nutricional foi realizado por meio da avaliação global subjetiva e pela aferição de medidas antropométicas. A diferença entre os grupos foi verificada pelo teste de Mann-Whitney e as associações, pelo χ^2^ de Pearson e correlação de Spearman.

**Resultados:**

A prevalência do risco de disfagia foi de 10,5%, sendo a faixa etária idosa um fator associado a esta condição. Pacientes em risco apresentaram valores inferiores de perímetro do braço e panturrilha, variáveis que se correlacionaram de forma inversa à pontuação do *Eating Assessment Tool* (EAT-10). A desnutrição foi identificada em 13,2% dos avaliados, segundo a avaliação global subjetiva, e em 15,2%, quando utilizado o índice de massa corporal.

**Conclusão:**

O rastreamento da disfagia e da desnutrição devem ser incorporados à rotina hospitalar, com o objetivo de evitar ou minimizar os prejuízos provocados por estas condições, especialmente nos idosos.

## INTRODUCTION

Dysphagia is defined as a swallowing difficulty in the passage of food from oral cavity to the stomach. This condition can be associated with symptoms such as regurgitation, tracheobronchial aspiration, retrosternal pain regardless of physical effort (related or not to feeding), pyrosis, hoarseness, hiccup and odynophagia.^(^ ^1^ ^)^Although this affection can occur at any age, aging, neurodegenerative disease and/or head and neck diseases are factors associated with this disease prevalence.^(^ ^2^ ^)^

This disease is underdiagnosed and its frequency is based on diagnosis methods. Dysphagia prevalence in general population ranges from 2% to 16%, values that can be higher than 40% in hospitalized patients.^(^ ^3^ ^)^ In hospital settings dysphagia is related with longer length of hospital stay, higher costs and higher mortality risk.^(^ ^4^ ^)^

Dysphagia is still associated with decrease in quality of life, aspiration pneumonia, dehydration, malnutrition, and social isolation.^(^ ^5^ ^)^ Clinical guidelines recommend that early identification of dysphagia risk and, in this sense, the instrument for its screening represent a practical alternative of low cost that enable to identify early cases in which a detailed evaluation is required.^(^ ^6^ ^)^

The Eating Assessment Tool (EAT-10) is a screening tool developed in the United States in 2008 using information collected from 482 patients. The EAT-10 is considered a valid and solid self-assessment tool to measures dysphagia risk and identifies individuals that need early multidisciplinary intervention.^(^ ^7^ ^)^ The instrument has 10 simple questions and provides information on functionality, emotional impact and physical symptoms that a swallowing problem can bring to an individual’s life.^(^ ^8^ ^)^ Gonçalves et al.,^(^ ^6^ ^)^ performed a cultural equivalence of EAT-10 in Brazilian Portuguese version without the need of changing or removal questions from the original protocol.

Older people have functional changes inherent to aging itself and, more frequently, diseases that increase the swallowing disorders risk.^(^ ^2^ ^)^ We also observed an association between women and presence of dysphagia, although there no consensus in literature exists about this subject.^(^ ^9^ ^)^ Malnutrition can also increase the frequency of dysphagia, because changes in consistence of food and the difficulty of ingestion can cause diet inadequacies. Higher score in the EAT-10 and indications of dysphagia risk were associated with change in nutritional status in older individuals.^(^ ^10^ ^)^ There is an estimation that 25% to 54% of hospitalized patients have some degree of malnutrition, and those with dysphagia have high risk of nutritional *deficits* and/or were already malnourished.^(^ ^11^ ^)^

Nutritional status is also an important aspect and should be evaluate in hospital settings. Well-recognized significant impacts that malnutrition impose are increase of risk of infections, complications, longer hospitalization, increase of hospital related costs and mortality, and reduction of immunology function. Nutritional diagnosis can be determined by subjective methods, as well as subjective global assessment (SGA), or also by evaluating objective anthropometric measures, mainly the body mass index (BMI). On the other hand, measurement of body perimeters such as calf circumference (CC) and arm circumference (AC) provide additional information on preservation of tissues, mainly muscle mass.^(^ ^12^ ^)^

## OBJECTIVE

To observe prevalence of dysphagia risk and associated factors in hospitalized patients, evaluate patients’ nutritional status using different methods, and correlate the status with the Eating Assessment Tool (EAT-10) score.

## METHODS

This was a cross-sectional study carried out in a philanthropic hospital using data collected from a institutional multidisciplinary database on nutritional therapy.

We analyzed data of adults and older individuals hospitalized between January to December 2014, and who underwent dysphagia screening, and assessment of nutritional status. We excluded from the study patients who were in the intensive care unit, pregnant and puerperal women. For dysphagia screening we used a culturally adapted to Brazil version of the EAT-10 questionnaire by Gonçalves et al.^(^ ^6^ ^)^ Patients with total score equal or greater than 3 points were classified as “at risk” for dysphagia.

Nutritional diagnosis was performed using SGA from Canada proposed by Detsky et al. The SGA assess changes in food consumption, gastrointestinal symptoms, functional capability, metabolic demands related with basis disease, and also perform physical exam involving muscle loss, adipocyte and edema. Using these parameters we classified participants as well-nurture (SGA-A) with suspicion of malnutrition or moderate malnutrition (SGA-B), and severe malnutrition (SGA-C).^(^ ^13^ ^)^

Anthropometrical evaluation includes weight measurement, CC, AC and knee height (KH).^(^ ^14^ ^)^ Weight measurement was done in electronic portable scale (Wiso W801^®^); perimeters were measure using inelastic tape (Cercorf^®^); and KH was measured with the aid of a stadiometer portable (Sanny^®^). Height was estimated for all evaluated, according to predictive formulas proposed by Chumlea et al.^(^ ^15^ ^)^

From measures of weight and height, we calculated BMI that was classified according to proposal by the World Health Organization to adults,^(^ ^16^ ^)^ and proposal by the Lipschitz,^(^ ^17^ ^)^ to older individuals (age equal or greater than 60 years). To CC assessment the adopted cutoff point was <31cm to indicate malnutrition.^(^ ^18^ ^)^ Adequacy of AC was determine using the equation: AC adequate (%) = [AC obtained (cm)) ÷ AC percentile 50] x 100, and classified by Blackburn et al.,^(^ ^19^ ^)^ We used percentile tables proposed in the United States by Frisancho,^(^ ^20^ ^)^ for individuals with no older than 74.9 years and by Kuczmarski et al.,^(^ ^21^ ^)^ for those who were even older.

Normal distribution of quantitative variables was determined using Kolmogorov-Sminorv test. Pearson’s χ^2^ test was used for comparison between proportions of dysphasia risk with sex, age and nutritional status based on BMI. Differences in anthropometric parameters between groups were evaluated using the Mann-Whitney test. Correlations between EAT-10 score and quantitative variables was verified according to Spearman’s correlation coefficient, considering that score in the EAT-10 did not present normal distribution (p<0.001). We adopted a p<0.05 as statistically significance in all analyses. Database was created using the Microsoft Excel^®^, and statistical analyses were performed using the Statistical Package for Social Science SPSS), version 21.

This study followed 466/2012 resolutions of National Health Council that defines guidelines and regulation norms for research involving human subjects. Our study was approved by the Ethical and Research Committee of the *Universidade Federal de Viçosa*, number 1.0000.31/2015, CAAE: 41396915.9.0000.5153.

## RESULTS

We evaluated data of 909 individuals with mean age of 54 years (standard deviation – SD 20.2 years). There was a predominance of women and adults patients. In relation to screening of dysphagia, 10.9% of evaluated individuals were classified as “at risk” (Table 1).

**Table 1:** Characteristics of the sample

Variable		n (%)
Sex		
	Male	419 (46.1)
	Female	490 (53.9)
Age range		
	Adults	534 (58.7)
	Older people*	375 (41.3)
EAT-10		
	No dysphagia risk	814 (89.5)
	With dysphagia risk	95 (10.5)
SGA		
	Well-nourished	789 (86.8)
	Suspicion of malnutrition or moderate malnutrition	81 (8.9)
	Severe malnutrition	39 (4.3)
BMI (n=902)		
	Thinness/low weight	137 (15.2)
	Eutrophia	408 (45.2)
	Overweight/obesity	357 (39.6)
CC (n=890), cm		
	<31	177 (19.9)
	≥31	713 (80.1)
AC (n=901)		
	Severe malnutrition (<70%)	37 (4.1)
	Moderate malnutrition (70-80%)	88 (9.8)
	Mild malnutrition (80-90%)	237 (26.3)
	Eutrophia (90-110%)	405 (45.0)
	Overweight (110-120%)	92 (10.2)
	Obesity (>120%)	42 (4.7)

* 60 years or more.

EAT-10: Eating Assessment Tool; SGA: subjective global assessment; BMI: body mass index; CC: calf circumference; AC: arm circumference.

Main reasons for hospitalization were gastrointestinal tract disease (15.1%), cardiovascular disease (13%), respiratory tract disorders (7.3%), fractures (9.8%) and neurologic diseases (5.7%). Associated morbidities found were seen in 27.8% of patients how had blood hypertension and in 16.2% of patients who had diabetes.

Based on SGA, 13.2% of individuals had some degree of malnutrition (B or C stages), 15.2% had low weight/thinness based on BMI, 19.9% had CC under the recommended and 40.2% had inadequacy of CC.

Comparison of age and anthropometric parameters, according to risk classification, revealed higher age and lower values of CC and AC in individuals who were at risk for dysphagia (Table 2).

**Table 2:** Comparison of objective variables according to risk for dysphagia

Variable	No risk		Risky		p value*
	Mean (SD)	Medium (IQR)	Mean (SD)	Medium (IQR)	
Age	53.14 (20.17)	53.00 (36.75-70.00)	62.73 (18.42)	64.00 (49.00-77.00)	<0.001
BMI	24.95 (4.87)	24.42 (21.50-27.72)	24.50 (5.87)	24.42 (20.94-27.88)	0.480
CC	34.21 (4.08)	34.45 (31.50-36.57)	32.97 (5.87)	32.85 (29.47-36.00)	0.008
AC	28.86 (6.23)	28.50 (26.00-31.40)	27.56 (4.50)	27.90 (24.45-30.92)	0.028

* Mann-Whitney test.

SD: standard deviation; IQR: interquartile range; BMI: body mass index; CC: calf circumference; AC: arm circumference.

Age range of older individuals was associated with high frequency of dysphagia risk (Table 3). Of those classified as “at risk”, 61% were aged 60 years or older.

**Table 3:** Associated factors to higher frequency of risk for dysphagia

Variable		No risk	Risky	p value*
		n (%)	n (%)	
Sex				
	Female	441 (90.0)	49 (10.0)	0.664
	Male	373 (89.0)	46 (11.0)	
Age range				
	Adults	497 (93.1)	37 (6.9)	<0.001
	Older people†	317 (84.5)	58 (15.5)	
BMI (n=902)				
	Thinness/low weight	115 (83.9)	22 (16.1)	0.064
	Eutrophia	370 (90.7)	38 (9.3)	
	Overweight/obesity	323 (90.5)	34 (9.5)	

* 60 years or older.

† Pearson’s χ^2^.

BMI: body mass index.

A direct, however, weak significant correlation was seen between score in the EAT-10 and in age. However, CC and AC had inversed correlations, although also very week, which indicated that higher the risk the greater the chance of dysphagia, this finding was associated with lower anthropometrical measures (Table 4).

**Table 4:** Correlation between *Eating Assessment Tool* (EAT-10) score and objective variables

Variable	ρ*	p value
Age	0.156	<0.001
Body mass index	-0.017	0.606
Calf circumference	-0.080	0.018
Arm circumference	- 0.078	0.019

* Spearman’s correlation coefficient.

## DISCUSSION

To the best of our knowledge, this study has the largest sample size reported in Brazil on evaluation of frequency of risk for dysphagia using the EAT-10 screening instrument in adults and hospitalized older individuals.

Prevalence of dysphagia risk found in the total sample and in older individuals was low than in studies that evaluated specific groups. A recent study^(^ ^10^ ^)^ evaluated 103 older individuals without previous history of dysphagia or swallowing problems. The EAT-10 used identified that 26.2% of individuals were at risk, and this condition was significantly associated with worsening in functional ability. Therefore, the tool was suggested as useful for screening of dysphagia, in addition the study highlighted the high prevalence they identified, because approximately one third of older individuals included in the study had suggestive sings of swallowing disorders.

Gálan Sánchez-Heredero et al.,^(^ ^22^ ^)^ evaluated an association between dysphagia risk according to the EAT-10, nutritional status and functional ability in 167 older inpatients in Spain. They showed an association between changes in swallowing with worsening of functional ability, higher frequency of comorbidities, aging, lower BMI, presence of nutritional risk and malnutrition. Prevalence of dysphagia risk and malnutrition were 30.8% and 15.4%, respectively, and, in patients at risk prevalence of nutritional problems increased up to 75%.^(^ ^22^ ^)^ Although in our study the association of EAT-10 with diagnosis using the BMI was not statistically significant, lower values of CC and AC in the risk group also suggest possible nutritional impact. Matsuo et al.,^(^ ^10^ ^)^ observed lower BMI and CC, in addition to reduction of hand grip strength and low calorie consumption of those with dysphagia. Calf circumference is considered a more sensitive muscle mass in older people and it indicates changes in lean mass that occur with aging and also decrease of physical activity.^(^ ^23^ ^)^ The AC is high correlated with body fat percentage and also constitute a good indicator for malnutrition assessment in older people.^(^ ^23^ ^,^ ^24^ ^)^

Age was the single variable significantly associated with risk for dysphagia. Older people are more susceptible to dysphagia because they often present more diseases associated with this condition, such as Parkinson’s disease, stroke, Alzheimer’s disease, amyotrophic lateral sclerosis, head and neck cancer, and dementia. In addition these individuals also present functional changes related with aging such as loss of strength and muscle tone, reduction of speed, precision and coordinator of movements, reduction of propulsion and esophagi peristaltic reflexes, and tooth loss.^(^ ^25^ ^)^

A study conducted by Freire et al.,^(^ ^26^ ^)^ in a hospital in Porto Alegre (RS), including specifically patients with Parkinson’s disease identified and associated dysphagia risk, by using the EAT-10, with changes in quality of life, mainly in relation to oral communication domain. In a study including 360 individuals with dysphagia, the EAT-10 score was observed as a predictor for occurrence of bronchoaspiration.^(^ ^27^ ^)^

There is a scarcity of reports in Brazil that used the EAT-10 in Brazil for comparison at national level. Although few studies exist, prevalence identified showed the importance of dysphagia screening in hospital settings. We highlight the need of validated instrument in Brazil in order to confirm applicability and reliability that were already confirmed in other countries.

Frequencies of malnutrition showed a high variability based on diagnosis method, and they ranged 13.2% using the SGA, and 40.2% according to AC. Regardless of the method, results showed relevance of nutritional *deficits* in hospital settings.

Frequency of malnutrition, based on SGA, was lower than the one found in a multicenter study conducted in Brazil by IBRANUTRI^(^ ^28^ ^)^ that included 4,000 inpatients; in this study almost half (48.1%) of individuals were identified with some degree of malnutrition. Prevalence based on BMI was similar in the study by Marcadenti et al.,^(^ ^29^ ^)^ that evaluated 445 individuals in a general hospital and identified malnutrition in 15.5% of the sample.

A study in Japan involving 874 individuals showed an independent association between malnutrition and dysphagia; a condition that increased the risk of morbidity, fragility and mortality. Changes in swallowing can be important predictors of malnutrition, mainly in older people.^(^ ^30^ ^)^

A limitation of this study was the cross-sectional design adopted that did not enable causal inferences related with dysphagia. Another limitation was the fact that anthropometrical measures were assessed by different evaluators. Although prior training was offered for measures standardization, there is the need to consider the possibility of interpersonal variability. A positive finding of this study was the prevalence of dysphagia risk and malnutrition in most of hospitalized patients. Health professional should be aware of the importance of early dysphagia screening that may influence significantly patients’ quality of life, morbimortality, recovery and prognosis.

## CONCLUSION

Assessment of risk for dysphagia using the EAT-10 score is a simple, quick and less expensive alternative to identify patients with swallowing problems. Prevalence found in our study emphasizes the importance of dysphagia screening in hospital settings, mainly among older patients. In addition to swallowing disorders, changes in nutritional status must be also routinely investigated by the adoption of adequate intervention measurement, monitoring and control.
